# Protective Role for Caspase-11 during Acute Experimental Murine Colitis

**DOI:** 10.4049/jimmunol.1400501

**Published:** 2014-12-29

**Authors:** Katarzyna Oficjalska, Mathilde Raverdeau, Gabriella Aviello, Siobhan C. Wade, Ana Hickey, Katherine M. Sheehan, Sinead C. Corr, Elaine W. Kay, Luke A. O’Neill, Kingston H. G. Mills, Emma M. Creagh

**Affiliations:** * School of Biochemistry and Immunology, Trinity Biomedical Sciences Institute, Trinity College Dublin, Dublin 2, Ireland;; † School of Medicine, Trinity Biomedical Sciences Institute, Trinity College Dublin, Dublin 2, Ireland; and; ‡ Royal College of Surgeons in Ireland and Beaumont Hospital, Dublin 9, Ireland

## Abstract

Activation of the noncanonical inflammasome, mediated by caspase-11, serves as an additional pathway for the production of the proinflammatory cytokines IL-1β and IL-18. Noncanonical inflammasome activity occurs during host defense against Gram-negative bacteria and in models of acute septic shock. We propose that the noncanonical inflammasome is activated in mice during acute intestinal inflammation elicited by dextran sodium sulfate (DSS), a model of experimental colitis. We find that caspase-11^−/−^ mice display enhanced susceptibility to DSS, because of impaired IL-18 production. The impaired IL-18 levels observed are shown to result in reduced intestinal epithelial cell proliferation and increased cell death. We also suggest that a novel type II IFN–dependent, type I IFN-TRIF–independent signaling pathway is required for in vivo caspase-11 production in intestinal epithelial cells during DSS colitis. Collectively, these data suggest that IFN-γ–mediated caspase-11 expression has a key role maintaining intestinal epithelial barrier integrity in vivo during experimentally induced acute colitis.

## Introduction

Activation of the prototypical proinflammatory caspase, caspase-1, by canonical inflammasome complexes results in the cleavage and secretion of IL-1β and IL-18 (1, 2). Caspase-11 is also an inflammatory caspase that is activated by a noncanonical inflammasome in response to Gram-negative bacteria (3, 4). Caspase-11 activation has recently been shown to have a key role in pyroptotic cell death, activated in response to intracellular pathogens (3, 5). Although pyroptosis was initially identified as a caspase-1–dependent process, caspase-11 is capable of inducing it independently of the canonical inflammasome (6, 7). These recent reports attributing caspase-11 with inflammatory and pyroptotic functions highlight the emerging importance of this inflammatory caspase during the innate immune response.

Once upregulated after TLR priming, caspase-11 is activated via the noncanonical inflammasome in response to the hexa-acyl lipid A moiety of bacterial LPS in the cytosol (5, 8). Activation of the noncanonical inflammasome represents an additional level of regulation for inflammasome-mediated caspase-1 activation after exposure to bacterial endotoxins. Although most of the studies elucidating the mechanism of noncanonical inflammasome activation have been carried out in vitro using bone marrow–derived macrophages (BMDMs), murine models of septic shock have confirmed that the noncanonical inflammasome drives lethal sepsis in vivo (5, 8), and studies with certain Gram-negative bacteria also reveal a role for the noncanonical inflammasome in vivo (9). This study aimed to investigate the contribution of caspase-11 to inflammatory processes that occur in vivo at distinct mucosal sites, such as within the intestine. Dextran sodium sulfate (DSS) administration to mice is widely used as a model of colitis, because it results in disruption of the intestinal epithelial barrier, exposing cells of the lamina propria to commensal bacteria and their products, such as LPS and peptidoglycan (10).

In this article, we describe a protective role for caspase-11 in vivo during acute DSS-induced intestinal inflammation, and demonstrate the involvement of caspase-11 in IL-18 production and maintenance of epithelial barrier integrity. Examination of the signaling events that lead to the upregulation and activation of caspase-11 in vivo suggest a novel requirement for type II IFNs during experimentally induced colitis. Thus, this study identifies a role for the noncanonical inflammasome in the control of mucosal integrity during acute colitis.

## Materials and Methods

### Mice

Casp11^−/−^ mice on the C57BL/6J background were obtained from J. Yuan’s laboratory (Harvard Medical School) and were subsequently backcrossed onto the C57BL/6J background for another eight generations. Heterozygous breeding pairs were used to generate wild type (WT) and Casp11^−/−^ littermates. Experiments were performed with 8-to 12-wk-old female mice bred under specific pathogen-free conditions, under license and approval of the local animal research ethics committee.

### Induction of colitis

Experimental colitis was induced in IFNAR^−/−^, IFN-γ^−/−^, TRIF^−/−^, Casp11^−/−^ mice, and WT littermates by adding 2% (w/v) DSS (m.w. 36,000–50,000; MP Biomedicals) to sterile drinking water for various numbers of days (3–7 d, as indicated). Fresh DSS solution was made and filter-sterilized every 3 d. The animals were weighed and monitored daily for signs of disease (weight loss, stool consistency, and rectal bleeding). On the final experimental day, mice were humanely sacrificed and colons were harvested. Colon length was measured as an indication of colonic inflammation. For the IL-18 rescue experiment, rIL-18 (R&D Systems) was i.p. injected at a concentration of 0.05 μg/mouse in 100 μl PBS for the first 7 consecutive days of the experiment.

### Histology

Sections from the distal colon of each mouse were analyzed using H&E staining. Colitis severity was assessed by a combined score of colon cellular infiltration (0–3, according to the extent of inflammation throughout the intestinal wall) and tissue disruption (0–3, according to the severity of mucosal and crypts damages) as described previously (11). The histological scoring was performed in a blinded fashion.

### Immunohistochemistry

The sections were deparaffinized in Histoclear (National Diagnostics) for 5 min, progressively rehydrated in decreasing concentrations of ethanol (100, 90, and 70% for 5 min each), and finally incubated in water for 5 min. The Ag retrieval process was performed by incubating the slides in boiling 0.01 M sodium citrate buffer (pH 6.0) for 10 min. The sections were then washed in PBS containing 0.1% Tween 20 and incubated overnight at 4°C with rabbit primary Ab proliferating cell nuclear Ag (PCNA; Abcam) at a dilution of 1:100. After several washes, the slides were incubated for 45 min with Alexa Fluor 488–conjugated goat anti-rabbit IgG (Invitrogen) at a dilution of 1:500. The sections were mounted using fluorescence mounting medium (DakoCytomation) containing DAPI for DNA staining and viewed on a point-scanning confocal microscope (FV1000; Olympus). Images were obtained and analyzed using Olympus FV-10 ASW viewer software. Quantitative fluorescence intensity measurement of PCNA-positive epithelial cells was analyzed in three specifically selected areas of colonic tissue per mouse using Imaris software.

### Cell death assay (TUNEL staining)

Apoptosis in distal colonic tissue sections was analyzed by fluorescence microscopy using an in situ cell death detection kit (Roche) according to the manufacturer’s protocol. For analysis, five random optical fields per colon were taken for the water treated and 2% (w/v) DSS-treated animals.

### In situ intestinal proliferation assay

Proliferating cells were detected by immunoperoxidase staining for thymidine analogue BrdU incorporation. In brief, BrdU (BD Biosciences) was dissolved in PBS at 1 mg/ml and injected i.p. at 50 mg/ml body weight. Distal colon tissues were collected from mice 3 h later, fixed in 10% (v/v) neutral-buffered formalin for 24 h, and embedded in paraffin. Immunohistochemistry was performed using an in situ BrdU staining kit (BD Biosciences). Tissues were counterstained with hematoxylin. The number of BrdU^+^ cells per 50 well-oriented crypts/mouse was analyzed.

### FITC-dextran assessment of intestinal permeability

Mice were gavaged with permeability tracer FITC-dextran (molecular mass, 4 kDa; FD4; Sigma-Aldrich) at a concentration 60 mg/100 g body weight. Four hours after gavage, blood was collected by cardiac puncture. Fluorescence of FITC-dextran in serum was measured on a FLUOstar OPTIMA Microplate Reader (BMG Labtech) at 490 nm excitation and 520 nm emission wavelengths. FITC-dextran concentration was determined from a standard curve generated by serial dilutions of FITC-dextran.

### Isolation of colonic epithelial cells

Colonic epithelial cells were isolated using the BD Cell Recovery Solution (VWR), as previously described (12). In brief, harvested colons were longitudinally sectioned, washed in ice-cold PBS, and cut into 3-cm-long fragments. Colon segments were incubated in a 5 ml ice-cold Cell Recovery Solution at 4°C for 4 h without agitation. Samples were then vortexed, centrifuged, and washed twice in ice-cold PBS. Next, the cell pellets were resuspended in RPMI 1640 medium (Invitrogen) and filtered through 100-μm cell strainers. Cell lysates were analyzed by Western blotting. Enrichment for colonic epithelial cells was determined by probing membranes with the epithelial cell–specific marker, cytokeratin-18.

### BMDMs culture and stimulation

BMDMs from WT and various knockout mice were cultured in DMEM with 10% (v/v) FBS and 20% (v/v) M-CSF–conditioned medium (L929 supernatants) for 9–11 d, then plated at ∼2 × 10^5^ cells/ml and cultured overnight. BMDMs were primed with LPS (200 ng/ml; Sigma-Aldrich) for 3 h and then stimulated with IFN-γ (60 ng/ml; R&D Systems) and IFN-β (500 IU/ml; PBL IFN Source) for 13 h. The following day, cell lysates were generated and samples were analyzed by Western blot.

### Cytokine measurements

To determine the cytokine amounts in colon tissue, we homogenized mechanically middle sections of the colons in tissue lysis buffer (1× PBS, 1% [v/v] NP-40 and protease inhibitor mixture; Roche). Colon homogenates were centrifuged at 14,000 rpm for 10 min at 4°C, and sample supernatants were used for cytokine concentration measurements using ELISA kits (R&D Systems/Biolegend) according to the manufacturer’s guidelines.

### Immunoblotting

Cell culture lysates and colon homogenates were lysed in radioimmunoprecipitation assay buffer (50 mM Tris, pH 8, 150 mM NaCl, 0.1% [w/v] SDS, 0.5% [w/v] sodium deoxycholate, and 1% (v/v) NP-40) supplemented with 5 mM EDTA and protease inhibitors (Sigma-Aldrich). Samples were clarified, denatured with 2× SDS loading buffer, and boiled for 5 min. A total of 40 μg protein lysate was fractionated on 12% SDS-PAGE, transferred to nitrocellulose, and probed with primary Abs to mouse caspase-11 (Sigma-Aldrich) and β-actin (Sigma-Aldrich).

### Statistical analysis

Data were analyzed using Prism 5 software (GraphPad). Error bars indicate SEM, as indicated. The unpaired one-tailed Student *t* test was used to compare the mean values between two groups. Statistical differences in mean values between more than two experimental groups were determined by two-way ANOVA followed by Bonferroni posttest. The *p* values <0.05 were considered significant.

## Results

### Increased susceptibility of caspase-11^−/−^ mice to DSS colitis

**FIGURE 1.**
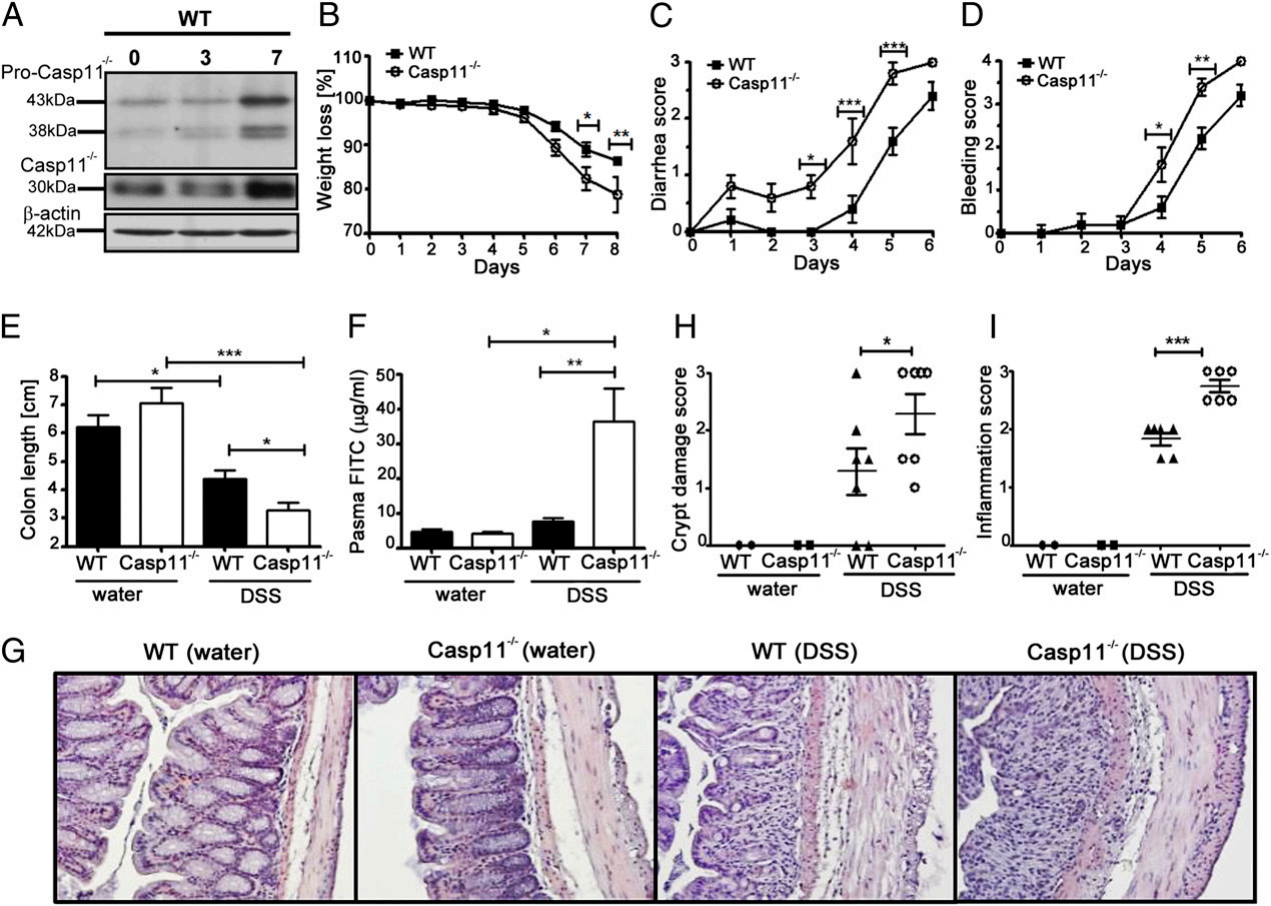
Protective role for caspase-11 during 2% DSS-induced colitis. (**A**) Western blot revealing caspase-11 expression in colon homogenates of C57BL/6 mice administered with 2% DSS in drinking water on days 0, 3, and 7. Each lane represents an individual mouse. Results are representative of three independent experiments. (**B**) Body-weight loss, (**C**) diarrhea score, (**D**) bleeding score, and (**E**) colon length measurements of WT and Casp11^−/−^ mice treated for 6 d with 2% DSS and for 2 subsequent days with regular drinking water. This experiment was repeated three times with similar results. Data represent mean ± SEM (2% DSS, *n* = 5; control, *n* = 2). (**F**) Plasma FITC-dextran concentrations of water and 2% DSS-treated WT and Casp11^−/−^ mice, 4 h after oral gavage of FITC-dextran (60 mg/100 g) on the last day of the experiment (day 7). Experiment was repeated twice with similar results. Data represent mean ± SEM (2% DSS, *n* = 6; control, *n* = 3). (**G**) Representative microscopic pictures of H&E-stained colon sections of water and 2% DSS-treated WT and Casp11^−/−^ from mice on the last day of the experiment (day 8). Original magnification ×200. (**H** and **I**) H&E-stained distal colonic tissues were assessed by semiquantitative scoring of tissue disruption (crypt damage score) and colon cellular infiltration (inflammation score). Data represent mean ± SEM (2% DSS, *n* = 6; control, *n* = 2). Statistical significance is indicated. *p < 0.05, **p < 0.01, ***p < 0.001.

To determine whether caspase-11 is involved in intestinal inflammation, we examined its expression levels during a model of acute colitis in WT C57BL6/J mice. Experimental colitis was induced by the administration of 2% DSS in drinking water (13). Colon homogenates from WT mice revealed that caspase-11 is robustly activated during the inflammatory phase of experimental colitis (Fig. 1A). Characterization of the noncanonical inflammasome led to the discovery that previously generated caspase-1–null mice (generated on the 129 strain) also lack a functional caspase-11 gene, making them caspase-1, caspase-11 double-knockout mice (3). In light of this discovery, the contribution of caspase-11 to the conflicting phenotypes that have been reported for caspase-1–null mice during DSS-induced colitis need to be re-examined, because capase-1 has been attributed with both protective and detrimental roles in the pathogenesis of colitis (14–16). Our findings in Fig. 1A suggest a role for caspase-11 during intestinal inflammation induced by DSS. Therefore, we addressed this hypothesis by examining experimentally induced acute colitis in caspase-11–deficient (Casp11^−/−^) mice, characterized in Supplemental Fig. 1. We found that Casp11^−/−^ mice had increased susceptibility to DSS-induced inflammation compared with WT littermate control mice, exhibiting more pronounced weight loss (Fig. 1B) and more severe and earlier incidences of diarrhea (Fig. 1C) and rectal bleeding (Fig. 1D) over the disease course. Assessment of colon length revealed more significant shortening in Casp11^−/−^ mice when compared with WT mice (Fig. 1E). Epithelial barrier permeability, as measured by FITC-dextran presence in serum, was also significantly higher in Casp11^−/−^ mice (Fig. 1F). The WT and Casp11^−/−^ mice, being littermates, should have identical microbiota. However, the DSS-colitis model was also performed using cohoused mice, which resulted in similar susceptibility of Casp11^−/−^ mice (Supplemental Fig. 2). This excludes any possibility that differences in microflora may account for the different susceptibilities observed between the two groups.

Histological analysis revealed increased inflammatory cell infiltration and mucosal damage in DSS-treated Casp11^−/−^ mice, with inflammation resulting in severe loss of crypt architecture (Fig. 1G–I). These results suggest that caspase-11 has a protective role in the intestine during DSS-induced colon inflammation. Similar phenotypes have been observed in caspase-1–deficient mice, and also in mice deficient in the canonical inflammasome components, NLRP3 and ASC (15, 17), suggesting that the noncanonical inflammasome is activated in vivo during acute DSS-colitis.

**FIGURE 2.**
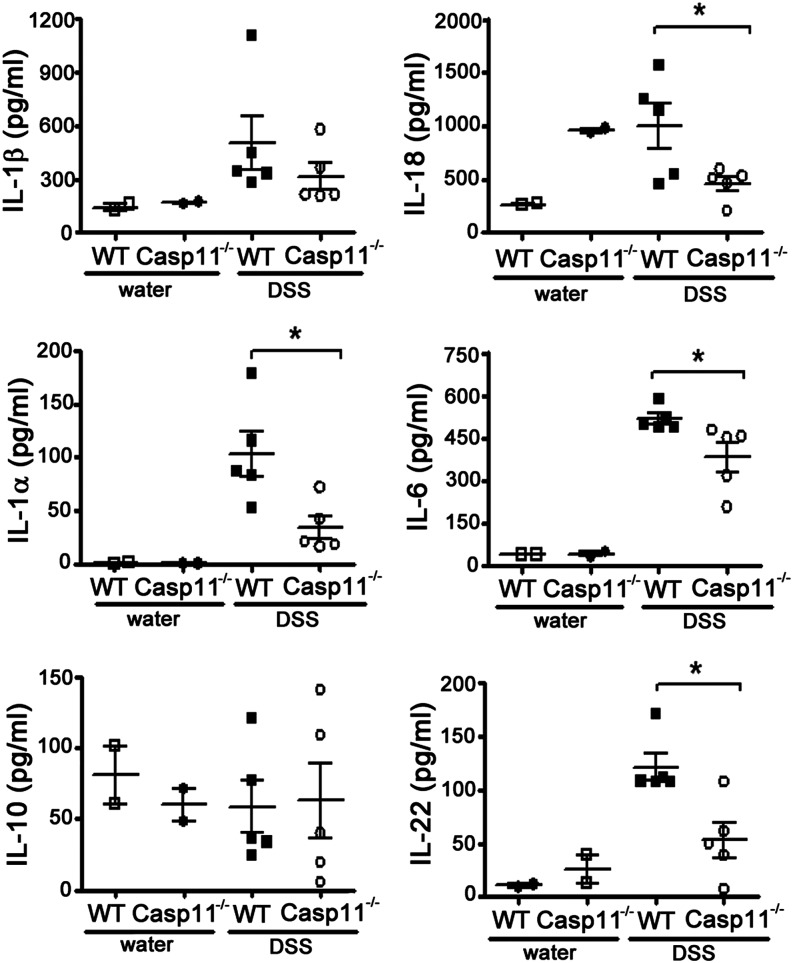
Cytokine levels in colons of DSS-treated mice. Cytokine (IL-1β, IL-18, IL-1α, IL-6, IL-10, and IL-22) levels in colon homogenates of DSS-treated WT and Casp11^−/−^ mice and their controls as measured by ELISA. This experiment was repeated three times with similar results. Data represent mean ± SEM of *n* = 5 mice and *n* = 2 for the control group. Statistical significance is indicated. *p < 0.05.

To determine the cytokine expression profiles of WT versus Casp11^−/−^ mice after exposure to DSS, we prepared colon homogenates from WT and Casp11^−/−^ mice 6 d after DSS administration, and cytokine concentrations were determined by ELISA. The data demonstrate significant defects in IL-18, IL-22, IL-1α (and to a lesser extent IL-6), but not in IL-1β or IL-10 production in Casp11^−/−^ mice (Fig. 2). IL-1α and the IL-18 precursor are both expressed constitutively in intestinal epithelial cells (IECs) throughout the gastrointestinal tract (18), whereas expression of the IL-1β precursor requires NF-κB–mediated priming, which may explain for the decreased levels of IL-1α and IL-18, but not IL-1β, observed in colons from Casp11^−/−^ mice. Interestingly, IL-18 levels are elevated in Casp11^−/−^ mice compared with WT mice at steady-state. This suggests that a caspase-11–independent mechanism, responsible for enhancing basal IL-18 expression, is activated in Casp11^−/−^ mice. However, during DSS colitis, intestinal upregulation of IL-18 expression appears to be regulated by caspase-11 (Fig. 2). Serum IL-18 levels from control and DSS-treated Casp11^−/−^ mice were not significantly different from those of WT mice, suggesting that the observed differences in IL-18 expression were confined to the intestine (Supplemental Fig. 3). Both IL-22 and IL-18 have important roles in IEC proliferation and epithelial barrier repair (19, 20); therefore, their decreased levels in Casp11^−/−^ mice during DSS colitis (Fig. 2) are consistent with the increased barrier permeability and severe mucosal damage observed in these mice (Fig. 1F, 1G).

### IL-18 rescues the colitis susceptibility phenotype of caspase-11^−/−^ mice

Inflammasome-mediated IL-18 production has previously been shown to have an essential role in intestinal epithelial barrier function during acute colitis; the enhanced susceptibility to colitis in caspase-1^−/−^, ASC^−/−^, and NLRP3^−/−^ mice could be reversed by the administration of IL-18 (15, 17).

**FIGURE 3.**
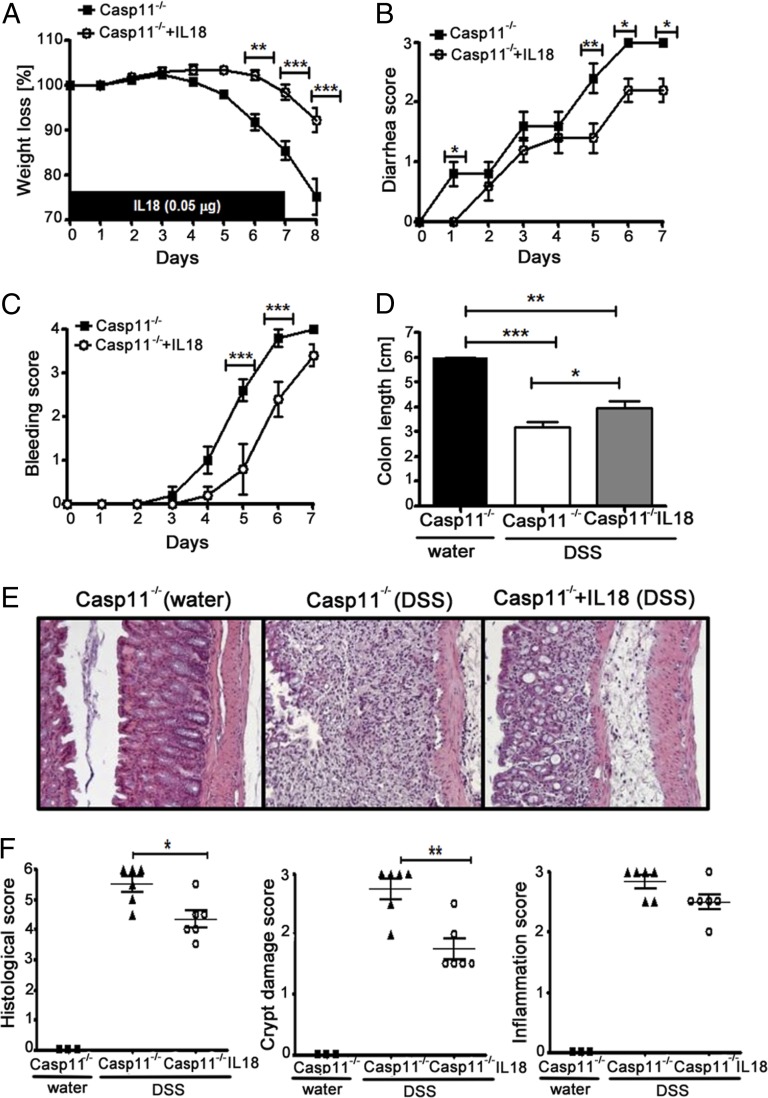
Exogenous IL-18 administration decreases the severity of DSS-induced colitis in Casp11^−/−^ mice. (**A**) Body-weight loss of Casp11^−/−^ mice ± IL-18. Casp11^−/−^ mice were i.p. injected with PBS or 0.05 μg rIL-18 for the first 7 d. Two percent DSS was administered for 6 d, followed by 2 d with regular drinking water. (**B**) Diarrhea score, (**C**) bleeding score, and (**D**) colon length measurements of Casp11^−/−^ mice ± IL-18 treated with DSS as in (A). Data represent mean ± SEM (2% DSS, *n* = 5; control, *n* = 3). This experiment was repeated twice with similar results. (**E**) Representative microscopic images of H&E-stained colon sections of water and 2% DSS-treated Casp11^−/−^ mice ± IL-18 on the last day of the experiment (day 8). Original magnification ×200. (**F**) H&E-stained distal colonic tissues were assessed by a combined histological score of tissue disruption (crypt damage score) and colon cellular infiltration (inflammation score). Data represent mean ± SEM (2% DSS, *n* = 6; control, *n* = 3). Statistical significance is indicated. *p < 0.05, **p < 0.01, ***p < 0.001.

To determine whether decreased IL-18 production was also responsible for the excessive DSS-induced colon damage in Casp11^−/−^ mice, we administered rIL-18 i.p. to mice during DSS-induced colitis. The results reveal that the severity of the caspase-11–null phenotype is rescued by administration of exogenous IL-18, which resulted in significantly less weight loss, diarrhea, rectal bleeding, and colon shortening than in PBS-treated Casp11^−/−^ mice (Fig. 3A–D). These findings suggest that the IL-18 deficiency observed in colon homogenates from Casp11^−/−^ mice (Fig. 2) contributes to their enhanced susceptibility to colitis. Histological analysis of H&E-stained colons revealed significant protection of crypt architecture in IL-18– compared with PBS-treated Casp11^−/−^ mice with DSS-induced colitis, which is consistent with a specific role for IL-18 in intestinal epithelial regeneration and repair in this disease model (Fig. 3E, 3F). Analysis of colon homogenates taken at day 8 revealed that concentrations of IL-18, IL-1α, and IL-6 remained unchanged after IL-18 administration (data not shown). Therefore, although the severity of disease was attenuated by exogenous administration of IL-18, any regulatory effects on endogenous cytokine levels were short-lived. Thus, the major protective effect of IL-18 on disease deterioration appears to be at the level of the epithelial barrier. Given the phenotypic similarities (increased susceptibility caused by deficient IL-18 production) between the Casp11^−/−^ mice and Casp1^−/−^, ASC^−/−^, and NLRP3^−/−^ mice (15, 17), these data strongly suggest a protective in vivo role for noncanonical inflammasome-mediated IL-18 expression during DSS-induced intestinal inflammation.

### Caspase-11–mediated IL-18 production results in IEC proliferation

**FIGURE 4.**
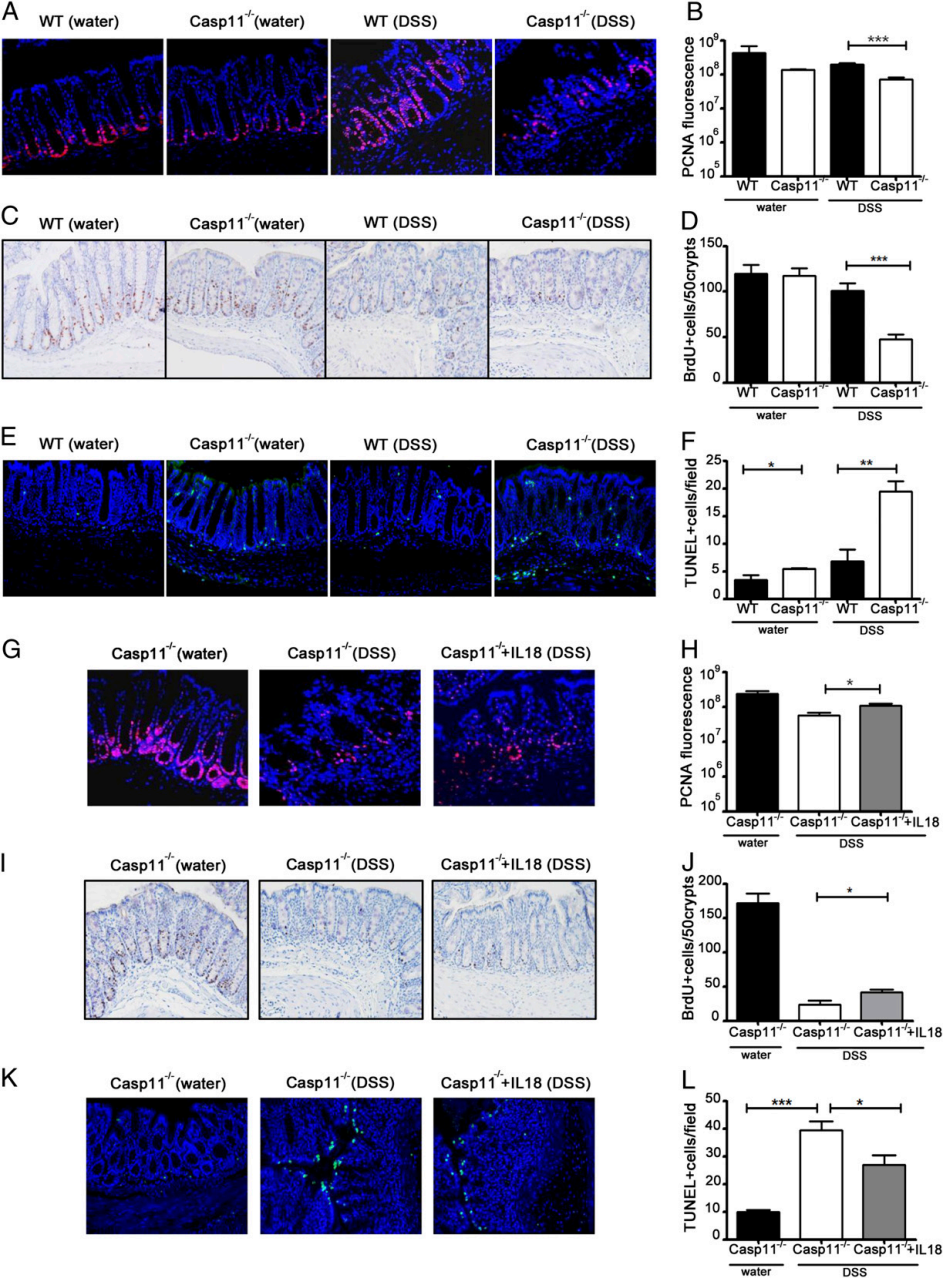
IL-18–induced IEC proliferation reduces the severity of DSS damage in Casp11^−/−^ mice. (**A**) Immunofluorescence staining of the proliferation marker (PCNA) from distal colon tissue sections of water and DSS-treated WT and Casp11^−/−^ mice and (**G**) Casp11^−/−^ ± IL-18 (day 8). Original magnification ×400. (**B** and **H**) Fluorescence quantification of PCNA^+^ cells shown in (A) and (G). On average, 30 crypts (3 fields of 10 crypts) were scored per mouse. Data represent mean ± SEM (2% DSS, *n* = 6; control, *n* = 3). (**C**) Immunohistochemical staining of the proliferation marker (BrdU) from distal colon tissue sections of water and DSS-treated WT and Casp11^−/−^ mice and (**I**) Casp11^−/−^ ± IL-18 (day 5). Original magnification ×200. (**D** and **J**) Quantification of BrdU^+^ cells shown in (C) and (I). On average, 50-well oriented crypts were scored per mouse. Data represent mean ± SEM (2% DSS, *n* = 6; control, *n* = 4). (**E**) Fluorescence staining of the TUNEL^+^ cells from distal colon tissue sections of water and DSS-treated WT and Casp11^−/−^ mice and (**K**) Casp11^−/−^ ± IL-18 (day 5). Original magnification ×200. (**F** and **L**) Quantification of TUNEL^+^ cells per field shown in (E) and (K). On average, five random optical fields were scored per mouse. Data represent mean ± SEM (2% DSS, *n* = 6; control, *n* = 4). Statistical significance is indicated. *p < 0.05, **p < 0.01, ***p < 0.001.

To investigate whether a defect in epithelial barrier maintenance and repair could explain the enhanced susceptibility of Casp11^−/−^ mice to acute colitis, we assessed IEC proliferation by staining for PCNA and 5-bromo-2-deoxyuridine (BrdU) incorporation into DNA. Colons from DSS-treated Casp11^−/−^ mice displayed significantly less IEC proliferation than those of WT mice (Fig. 4A–D). Similar results have been previously shown for DSS-treated NLRP3^−/−^ and Casp1^−/−^ mice, and thus the canonical inflammasome (15, 17). Epithelial cell death, a characteristic feature of colitis, was measured by TUNEL staining and shown to be significantly enhanced in DSS-treated Casp11^−/−^ mice and, to a lesser extent, at the steady-state (Fig. 4E, 4F). These findings suggest that caspase-11 controls mucosal integrity during acute colitis by increasing epithelial cell proliferation and inhibiting cell death.

To determine the involvement of IL-18 in driving IEC proliferation and inhibiting cell death in the Casp11^−/−^ colitis model, we also stained colon sections from the IL-18 rescue experiment (Fig. 3E) for PCNA, BrdU, and TUNEL. Our findings revealed that the IL-18 rescue phenotype of Casp11^−/−^ mice correlated with increased IEC proliferation (Fig. 4G–J) and decreased cell death (Fig. 4K, 4L). The data presented in this article suggest that caspase-11–mediated IL-18 production is required for IEC proliferation and mucosal repair after acute DSS-induced intestinal injury.

### DSS-colitis–induced caspase-11 upregulation is independent of TRIF

**FIGURE 5.**
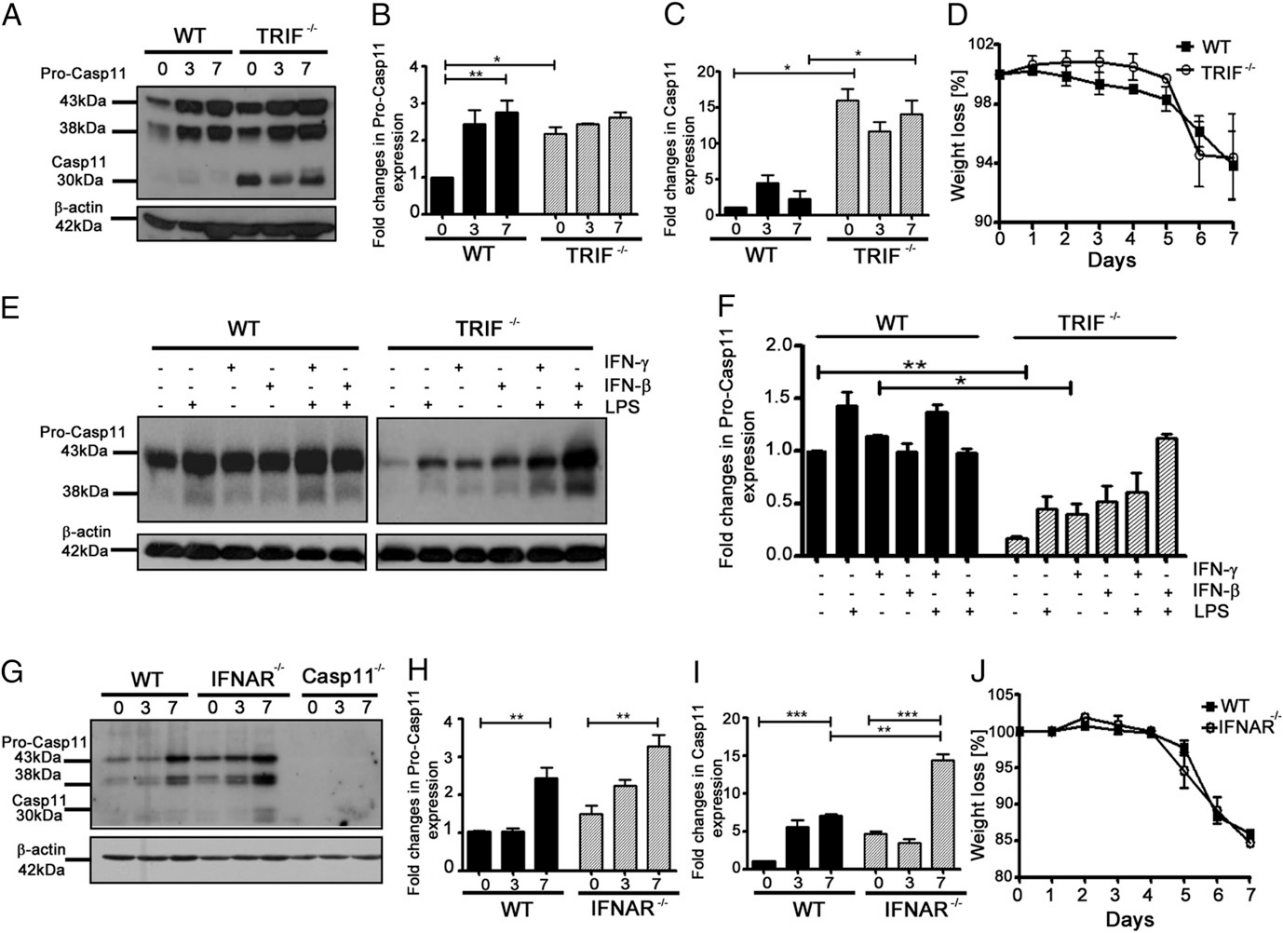
Mechanism of caspase-11 upregulation during DSS colitis. (**A**) Western blot showing caspase-11 expression in colon homogenates from WT and TRIF^−/−^ mice; (**G**) WT, IFNAR^−/−^, and Casp11^−/−^ mice treated with 2% DSS in drinking water for 0, 3, and 7 d. Each lane represents an individual mouse. Results are representative of two independent experiments. (**B**, **C**, **F**, **H**, and **I**) Relative densitometric analysis of indicated bands were performed using ImageJ software. (**D**) Body-weight loss of WT and TRIF^−/−^ mice and (**J**) WT and IFNAR ^−/−^ mice treated with 2% DSS in drinking water for 7 d and weighed daily. (D and J) Data represent mean ± SEM (2% DSS, *n* = 4). (**E**) Western blot detection of caspase-11 in WT and TRIF^−/−^ BMDMs stimulated as indicated. Results are representative of two independent experiments. Statistical significance is indicated. *p < 0.05, **p < 0.01, ***p < 0.001.

Previous studies have shown that noncanonical inflammasome activation after exposure to Gram-negative bacteria is mediated by TRIF and type I IFNs (4, 9). More recent data suggest that it may be the LPS-mediated priming of caspase-11, rather than its activation, that is dependent on TRIF and type I IFN signaling (5, 21). Because DSS administration causes epithelial damage and infiltration of commensal bacteria, a large proportion of which are Gram-negative, we hypothesized that TRIF and type I IFNs may also be responsible for the upregulation of caspase-11 in the colitis model. Contrary to our expectations, we did not find a defect in caspase-11 upregulation in colons from TRIF^−/−^ mice, but found an enhancement in caspase-11 activation in both untreated and DSS-treated mice (Fig. 5A–C). These findings suggest that TRIF is not required for caspase-11 upregulation or activation in vivo during DSS colitis. We did not observe significant differences in the susceptibilities of WT and TRIF-deficient mice to DSS-induced colitis (Fig. 5D). However, in the absence of TRIF, caspase-11 appears to be constitutively activated, suggesting that TRIF-mediated signaling events may even be involved in limiting caspase-11 and noncanonical inflammasome activation in vivo during DSS colitis. These data are at variance with previous in vitro studies, which used LPS-primed BMDMs infected with Gram-negative bacteria to demonstrate a clear requirement of TRIF for caspase-11 upregulation (4, 22). In agreement with these studies, we also observed a TRIF dependency for LPS-mediated upregulation of caspase-11 in BMDMs (Fig. 5E, 5F). This TRIF dependency can be overcome by the presence of IFN-γ or IFN-β (Fig. 5E, 5F), which has been previously demonstrated both in this context and in the context of restoring inflammatory responses after exposure to Gram-negative bacteria (4, 23). Our data highlight the additional signaling complexities of experimental disease models, such as DSS colitis, which cannot be recapitulated using LPS or specific Gram-negative bacterial infections either in vitro or in vivo.

We next examined the dependency of caspase-11 expression on type I IFNs. Similar to TRIF^−/−^ mice, caspase-11 was upregulated in IFNAR^−/−^ mice treated with DSS, and these mice also showed evidence of caspase-11 processing at day 7 (Fig. 5G–I). We found no significant difference between the susceptibilities of IFNAR^−/−^ and WT mice to DSS colitis, with similar weight-loss profiles (Fig. 5J). These data suggest that an alternative pathway to TRIF and type I IFN mediates the induction and activation of caspase-11 in vivo during this model of intestinal inflammation.

### Type II IFN signaling mediates caspase-11 upregulation during DSS colitis

**FIGURE 6.**
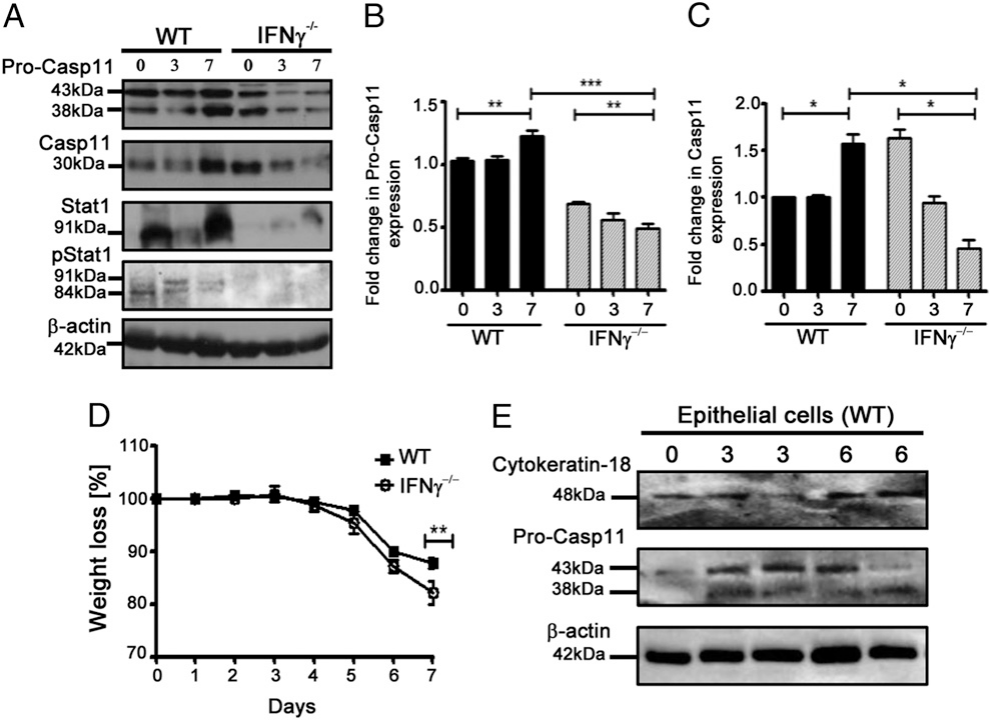
Type II IFN signaling is crucial for caspase-11 upregulation during DSS colitis. (**A**) Western blot showing caspase-11, STAT1, and phospho-STAT1 expression in colon homogenates from WT and IFN-γ^−/−^ mice treated with 2% DSS in drinking water for 0, 3, and 7 d. Each lane represents an individual mouse. Results are representative of two independent experiments. (**B** and **C**) Relative densitometric analysis of the individual band was performed using ImageJ software. Statistical significance is indicated. *p < 0.05, **p < 0.01, ***p < 0.001. (**D**) Body-weight loss of WT and IFN-γ^−/−^ mice treated with 2% DSS in drinking water for 7 d and weighed daily. Results are representative of two independent experiments. Data represent mean ± SEM (2% DSS, *n* = 8). **p < 0.01. (**E**) Western blot showing caspase-11 and cytokeratin-18 expression in epithelial cells isolated from WT mice treated with 2% DSS in drinking water for 0, 3, and 6 d.

Because caspase-11 has been shown to be responsive to both type I and II IFNs (4, 24), the possibility that IFN-γ may be involved in caspase-11 upregulation in mice during acute DSS colitis was explored. The data revealed that caspase-11 is not upregulated in IFN-γ^−/−^ mice in vivo after exposure to DSS (Fig. 6A–C). We also observed that, similar to Casp11^−/−^ mice, IFN-γ^−/−^ mice were more susceptible to DSS colitis (Fig. 6D). Caspase-11 levels appear to be normal in untreated IFN-γ^−/−^ mice (Fig. 6A), supporting the evidence that there are alternative pathways that control intestinal caspase-11 transcription, in response to distinct stimuli.

Similar to caspase-11, STAT1 expression and phosphorylation levels were enhanced in the colon during DSS colitis (Fig. 6A). As expected, STAT1 levels were significantly reduced in IFN-γ^−/−^ mice, suggesting that caspase-11 regulation during colitis may occur via a type II IFN-STAT1 signaling pathway. To gain further mechanistic insight into the regulation of caspase-11 in this model, we attempted to identify the cell types responsible for upregulating caspase-11 in the colon. Separation of epithelial and lamina propria cell layers from DSS-treated WT mice demonstrated that caspase-11 upregulation occurs in the IEC fraction after 3 and 6 d of DSS treatment (Fig. 6E). Taken together, these findings reveal a protective role for the noncanonical inflammasome in IECs during acute colitis.

## Discussion

The recent association of caspase-11 with a noncanonical inflammasome, combined with the knowledge that previous studies reporting a role for caspase-1 in colitis models of intestinal inflammation were likely carried out on Casp1,Casp11 double-knockout mice (14–16), led us to investigate the involvement of caspase-11 in intestinal inflammation. We observed decreased levels of IL-18 and IL-22 in DSS-treated Casp11^−/−^ mice, suggesting a defect in epithelial barrier repair (19, 20). IL-1α levels were also reduced. Caspase-11–mediated IL-1α production has recently been shown to stimulate neutrophil recruitment in vivo during bacterial infection of mice, suggesting that caspase-11 may also be responsible for recruiting neutrophils to sites of intestinal damage during DSS-induced colon inflammation (21), although this was not examined during our study.

Our findings link an increased DSS susceptibility of Casp11^−/−^ mice to reduced levels of IL-18 production, demonstrating that the susceptibility phenotype can be rescued via administration of IL-18 to Casp11^−/−^ mice. Previous studies have also linked caspase-1 deficiency to a reduction in intestinal IL-18 activity (14–16), which supports our findings and implicates the noncanonical inflammasome in DSS-mediated intestinal inflammation. We also identify an association among IL-18 production, IEC proliferation, and cell death after DSS treatment. In support of our findings, IL-18^−/−^ and IL-18R^−/−^ mice also have increased susceptibility to acute DSS colitis, highlighting the key role of this cytokine in intestinal regeneration and repair (25). NLRP6-inflammasome–mediated IL-18 secretion by IECs has been shown to prevent gut colonization by colitogenic microbiota (26), which further highlights the importance of IL-18 in protection and maintenance of epithelial barriers. The hypothesis that IL-18 has contrasting roles depending on the site of its expression (protective in IECs, but inflammatory at the level of the lamina propria) (19) provides an explanation for our findings during acute DSS damage, which primarily affects IECs. Furthermore, this study demonstrates that caspase-11 upregulation during DSS colitis occurs in IECs.

Caspase-11 appears to be regulated by a type II IFN–mediated pathway during DSS colitis and is totally independent of TRIF/IFNAR in this context. However, LPS-stimulated macrophages from TRIF^−/−^ mice were also shown, by us and others, to have impaired caspase-11 expression levels (4, 22), highlighting a context-dependent role for type I or II IFN in the regulation of caspase-11 activity. In support of this conclusion, it has been shown that IFN-γR^−/−^, but not IFNAR^−/−^, BMDMs are still capable of processing caspase-1 and caspase-11 postinfection with certain Gram-negative bacteria (22). However, during *Legionella pneumophila* infection, caspase-11 appears to be upregulated and activated independently of IFNAR and TRIF (21).

The caspase-11 promoter region contains a number of putative transcription factor binding sites, including partially overlapping κB and STAT sites, which were previously shown to synergistically regulate caspase-11 expression via LPS and IFN-γ in vitro (24). We have also observed synergistic upregulation of caspase-11 by IFN-γ and LPS in WT and TRIF^−/−^ BMDMs (Fig. 5E). A recent study has shown that IFN-γ–activated STAT1 synergistically enhances TLR-induced transcription of proinflammatory cytokines through epigenetic priming mechanisms (27). These findings suggest that IFN-γ may be required to directly prime the caspase-11 gene in vivo before it can be robustly upregulated in response to microbial patterns (such as LPS and other TLR ligands) encountered during colitis. If this hypothesis is correct, it suggests that the JAK-STAT1 pathway may be a potential target in the management of colitis.

Our study reveals an in vivo role for caspase-11 during intestinal inflammation in the DSS-induced acute colitis model. Our findings are consistent with a recent report that Casp11^−/−^ mice have increased susceptibility to acute experimental colitis and focused on the role of gut microbiota (28). Our study links caspase-11–mediated IL-18 production to the increased susceptibility of Casp11^−/−^ mice to colitis, suggesting the involvement of noncanonical inflammasomes in IEC proliferation, inhibition of cell death, and epithelial barrier function. We also propose a novel type II IFN–mediated signaling pathway leading to the upregulation and activation of intestinal caspase-11 in IECs in vivo. These findings highlight the emerging importance of noncanonical inflammasome activation in innate immunity and chronic inflammatory diseases, such as inflammatory bowel disease.

## Supplementary Material

Data Supplement
