# Supplementary material for: Protective Role for Caspase-11 during Acute Experimental Murine Colitis
Source: J Immunol. 2014 Dec 29;194(3):1252–60. doi: 10.4049/jimmunol.1400501 (PMC4298125; doi:10.4049/jimmunol.1400501)
Supplement: Data Supplement [file JI_1400501.zip › JI_1400501_Supplemental_Figures_1.pdf]

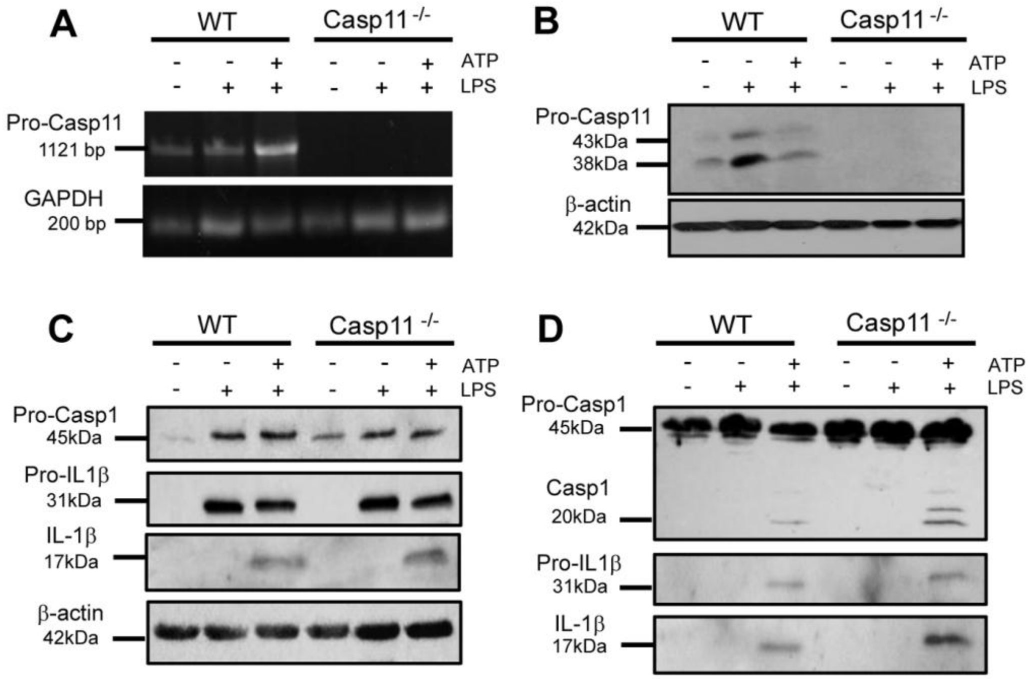

FIGURE S1: Examination of the canonical inflammasome pathway in Caspase-11 knockout mice.

(A) RT-PCR and **(B)** Western blot detection of caspase-11 in WT and Casp11$^{-/-}$ BMDMs plated at ~ 2 x 10^6 cells/ml and primed with LPS (1 µg/ml) for 4 h, followed by ATP (5mM) stimulation for 30mins.

(C) Western Blot detection of procaspase-1, pro-IL-1β, cleaved caspase-1 and IL-1β in the lysates, and **(D)** in the supernatants of LPS (1 µg/ml) primed BMDMs for 8 h, followed by ATP (5mM) stimulation for 30mins. For **(A)** GAPDH and for (**B, C** and **D**) β-actin was used as loading control. Results are representative of two independent experiments.

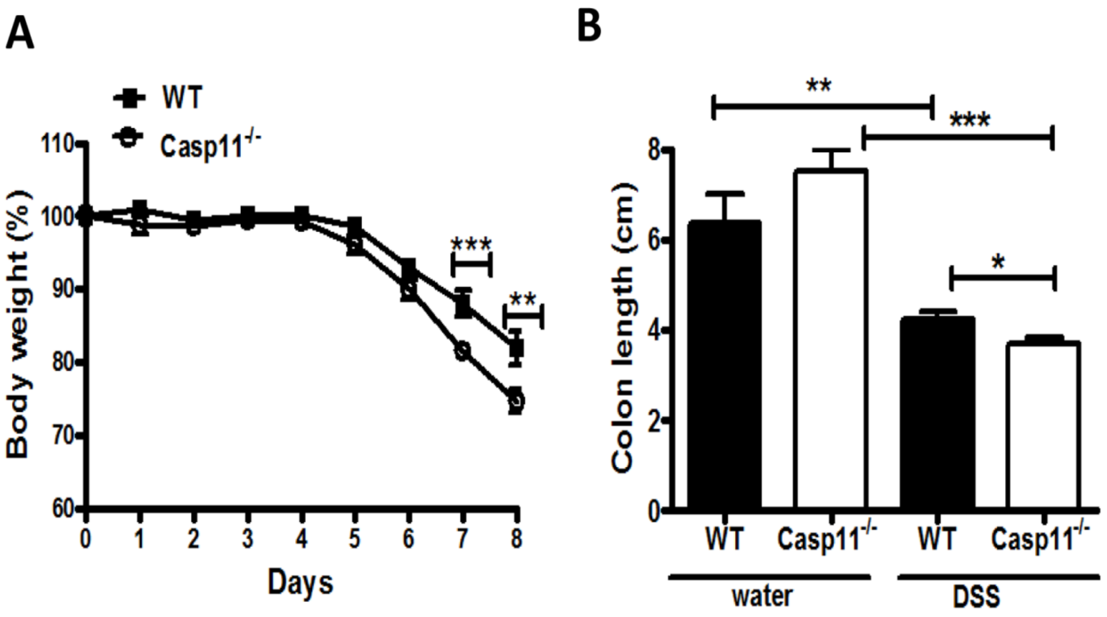

FIGURE S2: The microbiota of Casp-11$^{-/-}$ mice does not account for their hypersensitivity to DSS-induced colitis.

(**A**) Body weight loss and **(B)** colon length measurements of WT and Casp11$^{-/-}$ mice co-housed for 2 weeks before treatment with 2% DSS for 6 days, followed by 2 days with regular drinking water. Data represent mean ±SEM, (2% DSS, n=5; control, n=2). This experiment was repeated with similar results. Statistical significance is indicated; *p<0.05, **p<0.01, ***p<0.001.

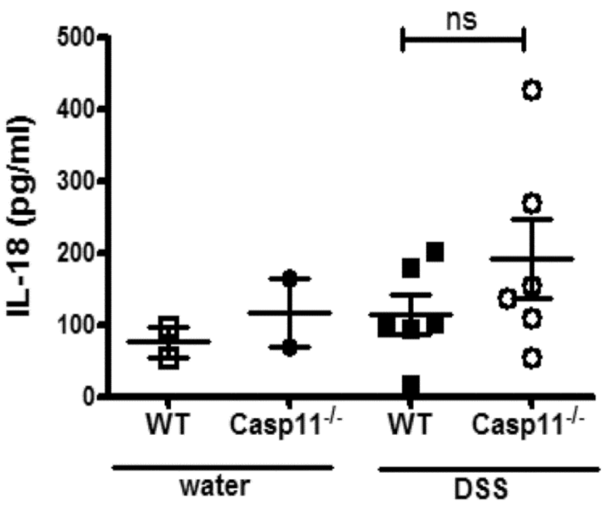

FIGURE S3: Levels of IL-18 in serum during DSS-induced colitis.

Systemic release of IL-18 in the serum of DSS treated WT and Casp11$^{-/-}$ mice and their controls on the last day of the experiment (day 7) measured by ELISA. Data represent mean ±SEM, (2% DSS, n=6; control, n=2).
